# *rbcL* gene in global molecular data repository

**DOI:** 10.1016/j.dib.2022.108090

**Published:** 2022-03-23

**Authors:** Conrad Omonhinmin, Chinedu Onuselogu

**Affiliations:** Department of Biological Science Biotechnology Cluster, College of Science and Technology, Covenant University, Canaan land Ota, Ogun State, Nigeria

**Keywords:** *rbcL* gene, Evolutionary, Biogeography, Phylogeny, Molecular repository

## Abstract

The ribulose-bisphosphate carboxylase (*rbcL*) gene sequence data in the molecular data repository has been increasing significantly, over the years with contributions from different parts of the world. The abundance of the gene has enhanced its applications in several ways. Bulk records were obtained from National Center for Biotechnology Information (NCBI) GenBank database using the entrez efetch utility as implemented in the Biopython package version 1.77. Records corresponding to the following keywords “*rbcL* AND plants [filter] AND biomol_genomic [PROP] AND is _nuccore [filter]” were created. Generated records were cleaned and then further analysed using the code file in the supplementary materials. Country information was obtained by searching reference information for matches to countries present in the pycountry package. Where no match was found, null was returned. This data article contains information about the plant family and species whose *rbcL* gene sequence has been deposited on the NCBI and regions of the world that has contributed to the *rbcL* repository growth. This data can be used to analyse the intra and inter family relatedness of plant and compare with existing relationships the molecular characterization of plants, evolutionary relationship studies, inferring biogeography origin of plant.

## Specifications Table

**Table:** 

Subject	Biological sciences
Specific subject area	Molecular phylogenetics, Phylogeny and Evolution
Type of data	Text, Table, Chart, Figure
How data were acquired	Biopython package version 1.77. was used to retrieve the *rbcL* gene sequence data from the NCBI GenBank. The written code used for retrieving the data from the NCBI GenBank can be assessed in the supplementary materials.
Data format	Raw, Analysed and Filtered.
Description of data collection	Bulk data were obtained from NCBI GenBank database using the entrez efetch utility as implemented in the Biopython package version 1.77. Datasets that do not have the matching words *rbcL*, Plant and DNA were filtered off from the data leaving behind data with the keywords *rbcL,* plant and DNA.
Data source location	The data was obtained from the NCBI GenBank database.
Data accessibility	With the article.
Repository name	Mendeley Data
Data identification number	10.17632/wdmtpnwsrn.1
Direct link to the dataset:	http://www.rbcLGeneinGlobalMolecularDataRepository.com

## Values of the Data

• This data present information of plant species, phylum, and family for which *rbcL* gene sequence have been deposited on NCBI GenBank.
• Molecular systematics can use the data to renew the relatedness of plants both within and between families as well as compare with existing relationships.
• This data is useful in the following field: molecular characterization of plants, evolutionary relationship studies, inferring biogeography, origin of plant, codon bias usage profile, protein structure analysis, ecological preference studies.
• This data can be used to determine the pattern of growth of *rbcL* gene sequence from different regions in the molecular repository.
• This data shows the least explore plant species and the need for exploitation.

## 1 Data Description

**Fig. 1:**
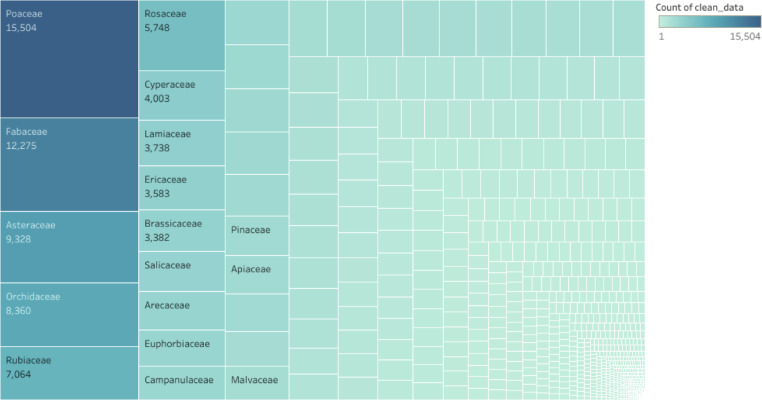
Most studied plant families with *rbcL* gene sequence in GenBank. *The numbers indicate the number of species in each family with *rbcL* gene deposited on NCBI GenBank. *NB: The study discovered a total number of 808 plant families with *rbcL* gene sequence submitted on the NCBI GenBank making it difficult to include all the families in the tree map in Fig. 1, hence the plant families with the most *rbcL* gene submission are mentioned in Fig. 1.

**Fig. 2:**
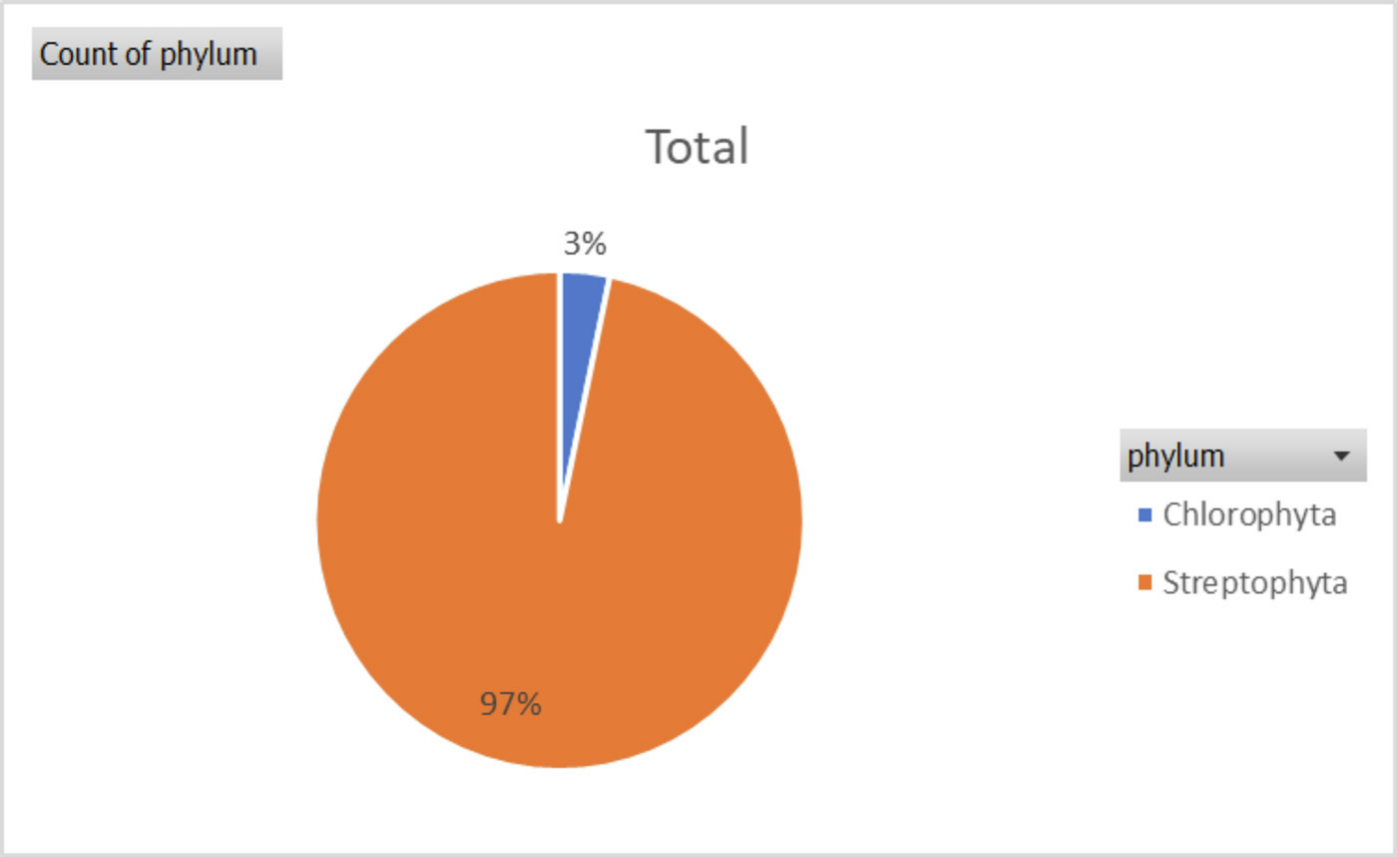
Percentage of plant phyla with *rbcL* gene data deposited on GenBank.

**Fig. 3:**
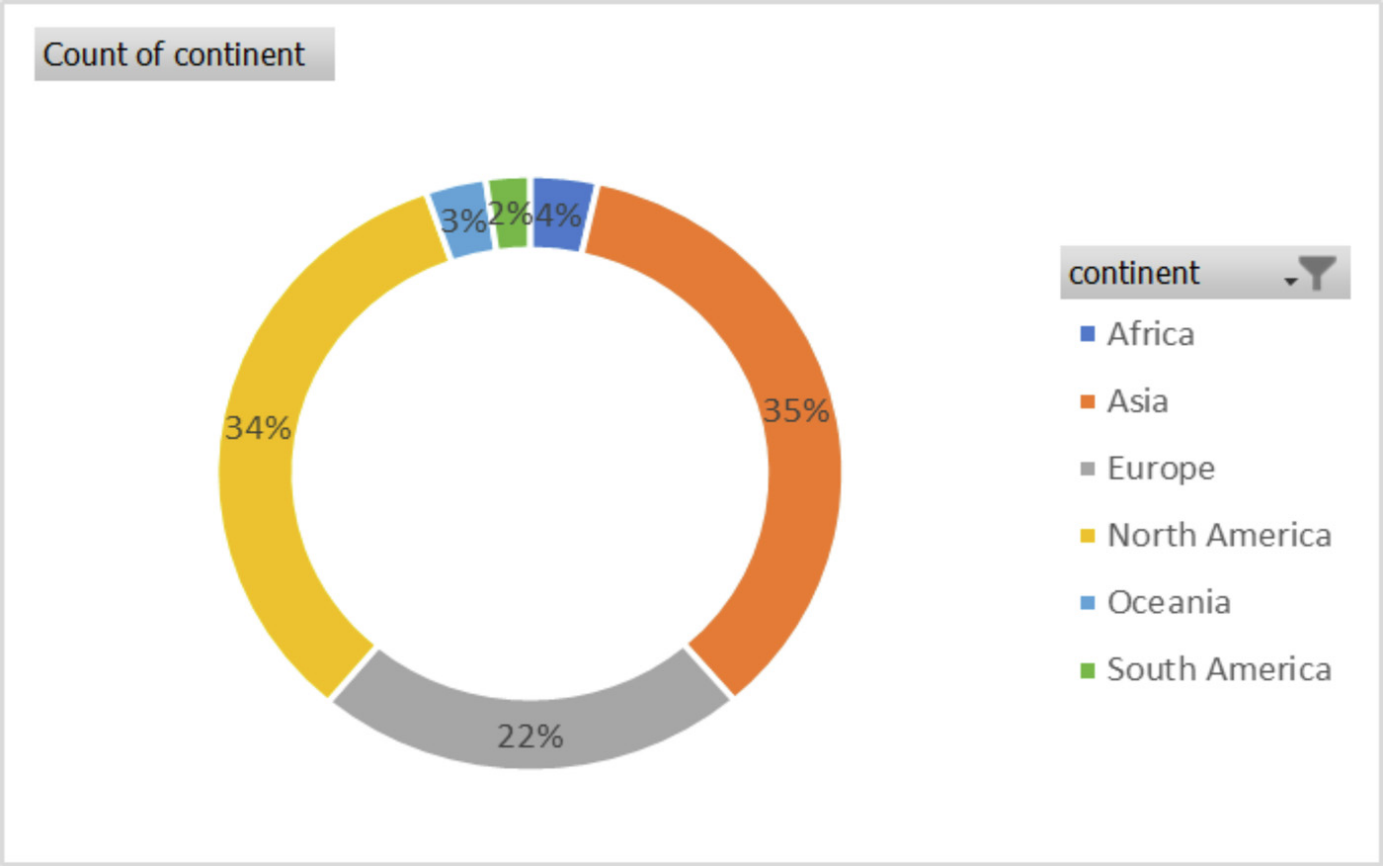
Percentage of *rbcL* sequences contribution from different regions.

**Fig. 4:**
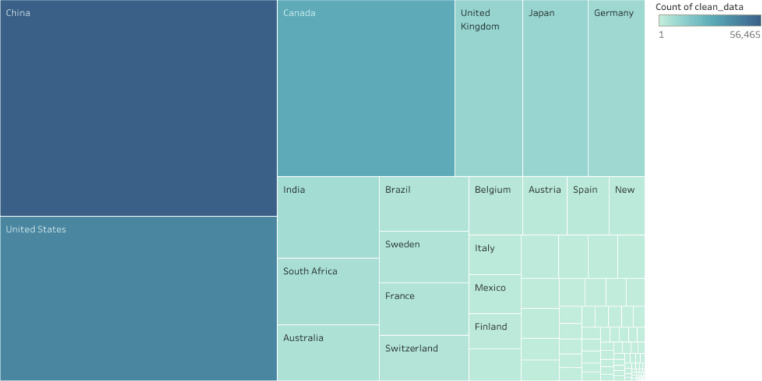
Countries with higher submissions of *rbcL* sequences on the GenBank repository.

**Fig. 5:**
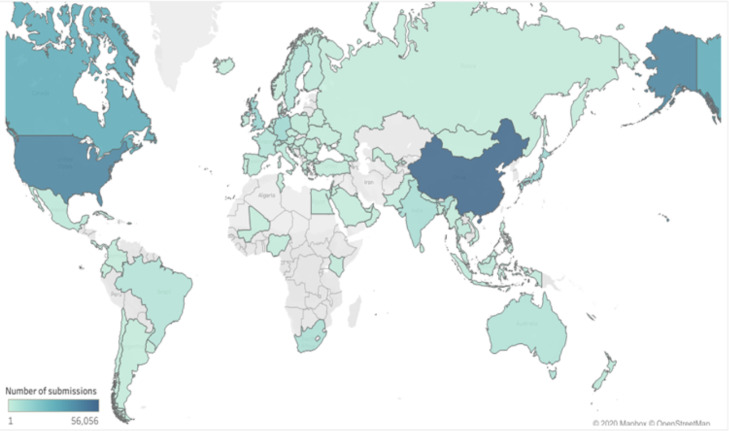
Map showing global concentration of *rbcL* sequence contribution to GenBank repository. * Regions with dark blue has higher contribution of *rbcL* gene sequence on the NCBI GenBank.

The data in this article gives an overview of the total number of plant species, families, with *rbcL* gene sequence in the GenBank and the regions that has contributed to the growth of the *rbcL* sequence in the repository. The sequence data of the *rbcL* gene are used for renewal of phylogenies among the seed plants [1]. The *rbcL* gene is preferred among other plant genes for phylogenetic studies due to its slower rate of evolutionary changes and the lowest divergence among the plastid genes in flowering plants [2,3]. [4] described the suitability of the gene for solving intergeneric and interspecific relationship and no difficulties of alignment. Some of the applications of the gene in the molecular investigations of plant species include: tracing of the molecular origin of plants [5], the biogeography origin of plants [6]. The datasets used, in the study was collected as a secondary data and the Bio python code written for data collection can be assessed as Supplementary data, the *rbcL* gene data used was obtained from the first report till 2020. Fig. 1, shows the most studied plant families with *rbcL* gene on the GenBank. Fig. 2; shows plant phyla with *rbcL* gene sequence and the extent to which the sequences have been utilized for *rbcL* related studies. The continents with *rbcL* sequence submission and the percentage of contribution to the GenBank is represented in Figs 3, 4 and 5 shows countries with higher *rbcL* sequence submission on GenBank. The plant species and other species with *rbcL* gene sequence can be assessed in the supplementary materials.

## 2 Experimental Design, Materials and Methods

Bulk records were obtained for NCBI GenBank database using the entrez efetch utility as implemented in the Biopython package version 1.77. Records corresponding to the following keywords “*rbcL* AND plants[filter] AND biomol_genomic[PROP] AND is_nuccore[filter]” were obtained. Obtained records were cleaned and then further analysed using the codes files in the supplementary material. Country information was obtained by searching reference information for matches to countries present in the pycountry package. Where no match was found, null was returned.

## CRedit Author Statement

**Conrad Omonhinmin:** Conceptualization, Methodology, Validation and Supervision; **Chinedu Onuselogu:** Data curation, Investigation, software, Reviewing and Editing, Writing-Original draft preparation.

## Declaration of Competing Interest

The authors declare that they have no known competing financial interests or personal relationships which have or could be perceived to have influenced the work reported in this article.

## Data Availability

rbcL Gene in Global Molecular Data Repository (Original data) (Mendeley Data).

## Appendix Supplementary materials

Image, application 1

Image, application 2

Image, application 3
